# Electrode and electrolyte configurations for low frequency motion energy harvesting based on reverse electrowetting

**DOI:** 10.1038/s41598-021-84414-3

**Published:** 2021-03-03

**Authors:** Pashupati R. Adhikari, Nishat T. Tasneem, Russell C. Reid, Ifana Mahbub

**Affiliations:** 1 Department of Mechanical and Energy Engineering, University of North Texas, 3940 N Elm St, Suite F101, Denton, TX 76207 USA; 2 Department of Electrical Engineering, University of North Texas, Denton, TX 76207 USA; 3 Department of Engineering, Dixie State University, Saint George, UT 84770 USA

**Keywords:** Energy science and technology, Energy harvesting, Devices for energy harvesting

## Abstract

Increasing demand for self-powered wearable sensors has spurred an urgent need to develop energy harvesting systems that can reliably and sufficiently power these devices. Within the last decade, reverse electrowetting-on-dielectric (REWOD)-based mechanical motion energy harvesting has been developed, where an electrolyte is modulated (repeatedly squeezed) between two dissimilar electrodes under an externally applied mechanical force to generate an AC current. In this work, we explored various combinations of electrolyte concentrations, dielectrics, and dielectric thicknesses to generate maximum output power employing REWOD energy harvester. With the objective of implementing a fully self-powered wearable sensor, a “zero applied-bias-voltage” approach was adopted. Three different concentrations of sodium chloride aqueous solutions (NaCl-0.1 M, NaCl-0.5 M, and NaCl-1.0 M) were used as electrolytes. Likewise, electrodes were fabricated with three different dielectric thicknesses (100 nm, 150 nm, and 200 nm) of Al_2_O_3_ and SiO_2_ with an additional layer of CYTOP for surface hydrophobicity. The REWOD energy harvester and its electrode–electrolyte layers were modeled using lumped components that include a resistor, a capacitor, and a current source representing the harvester. Without using any external bias voltage, AC current generation with a power density of 53.3 nW/cm^2^ was demonstrated at an external excitation frequency of 3 Hz with an optimal external load. The experimental results were analytically verified using the derived theoretical model. Superior performance of the harvester in terms of the figure-of-merit comparing previously reported works is demonstrated. The novelty of this work lies in the combination of an analytical modeling method and experimental validation that together can be used to increase the REWOD harvested power extensively without requiring any external bias voltage.

## Introduction

Trends in human health monitoring of physical activities and medical diagnostics have significantly enhanced over the last few decades^1–3^. Wearable and implantable sensors are used to monitor various health activities in real-time, which provide meaningful insights to the clinicians. The popularity of wearable technology has increased because of the usefulness of continuous human vital sign monitoring during day-to-day life activities such as walking, running, sports activities, and in clinical environments^4^. Traditionally, wearable sensors are powered using batteries that limit the longevity of the device due to the need for frequent battery replacement, which directly affects the device performance and reliability. Moreover, batteries impose a roadblock to device miniaturization and are, furthermore, related to certain safety issues, such as battery explosion or electrolyte leakage^5^. Therefore, there is an urgent need of developing energy harvesters that are able to reliably and sufficiently power such sensors. Among many ambient energy harvesting technologies that have emerged over the last several years, piezoelectric energy harvesters (PEH), triboelectric nanogenerators (TENG), electromagnetic energy harvesters, and vibration-based energy harvesters are well established^6,7^. Among these energy harvesting technologies, TENG has been demonstrated to efficiently perform at lower frequency range (0.25–5 Hz) which is the typical frequency range for several human motion activities^8,9^. However, TENGs require electrodes to undergo continuous solid–solid friction generating a large amount of heat, directly affecting the lifetime and reliability of the energy harvester^10^. Even though efforts have been made to minimize the frictional heat generation by introducing liquid and conductive cushioning materials between electrodes, the underlying principle by which TENG operates, frictional phenomenon is unavoidable thus imposing an obstacle to this technology^11,12^. While much progress has been made in the other energy harvesting technologies, most of them significantly suffer when it comes to harvesting energy from the low-frequency range (0.25–5 Hz). In addition, piezoelectric energy harvesting technique requires electrodes to undergo continuous material strain resulting in material degradation, directly affecting the lifetime and reliability of the energy harvester^13^. Electromagnetic energy harvesters are not ideal to power wearable sensors used in human health monitoring applications due to the possible safety issues arising from electromagnetic radiation. Federal Communication Commission (FCC) regulations limit electromagnetic device radiation to 1.6 W/kg to minimize energy absorbed by the human body, which could arise while using the electromagnetic energy harvesters^14^. Besides, electromagnetic energy harvesters require higher frequency input (> 100 Hz) to operate for optimal performance^15^. Piezoelectric energy harvesters have been used in various applications as a reliable source of power. However, their optimal performance typically occurs at resonant frequencies that are much higher than the typical human motion frequency range, thus making them unsuitable for self-powered human motion sensors^16,17^. Owing to the limitations of the existing energy harvesting technologies towards harvesting energy from human motion activities, there is an immediate need for an energy harvesting technology capable of operating efficiently at lower frequencies (< 5 Hz) with a longer lifetime.

Within the last decade, a new approach to electrostatic energy harvesting, known as reverse electrowetting-on-dielectric (REWOD) has emerged^18^. Unlike many other energy harvesting technologies, REWOD has been demonstrated to operate efficiently at a low mechanical frequency range because of its independence from the resonance of the solid structures^19–21^. The REWOD mechanism is the opposite of electrowetting-on-dielectric (EWOD), where an applied voltage results in a mechanical motion of liquid droplet(s). In REWOD, an applied mechanical force results in a voltage due to the increase in the electrical capacitance. The occurrence of capacitance in the electrode–electrolyte interface in REWOD is the result of the capacitance from both the electrical double layer (EDL) and the dielectric insulator. REWOD models that are facilitated with an external bias voltage have almost no significant contribution from the EDL due to the bias-voltage induced high capacitance. However, in the absence of the bias-voltage, capacitance due to both the EDL and the dielectric insulator is the effective “capacitance” and is referred to as such from here on throughout this paper.

**Figure 1:**
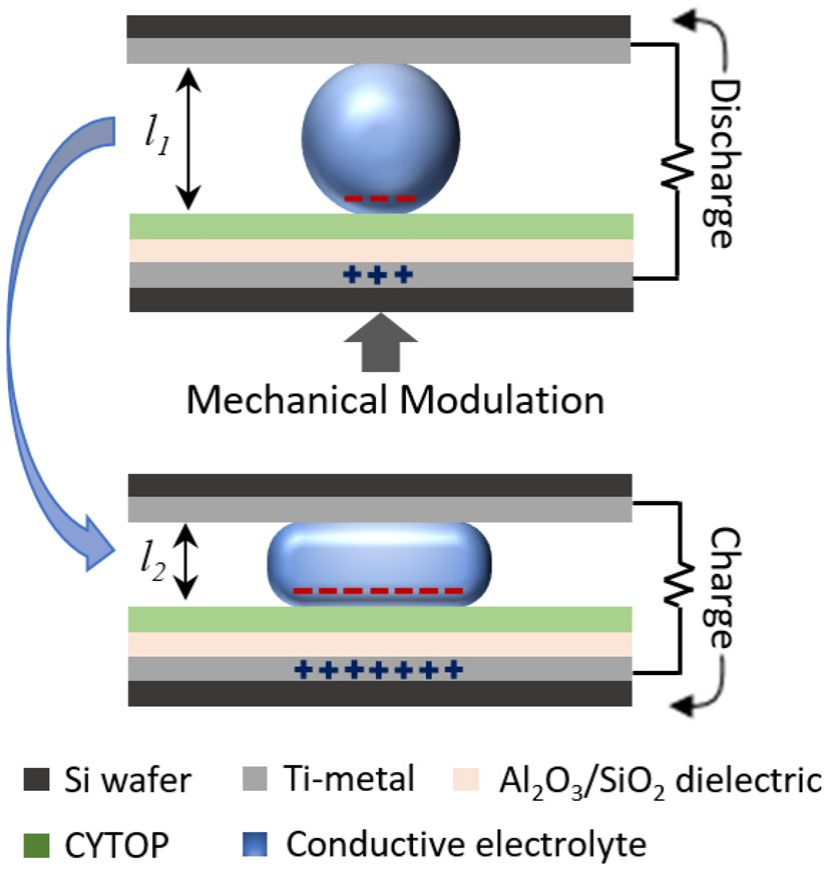
Working principle of REWOD energy harvesting without any applied bias voltage. Maximum mechanical displacement of electrodes during modulation, *Δl* is given as: *Δl* = *l*_*1*_* − l*_*2*_.

Figure 1 illustrates an example of the REWOD configuration wherein the top electrode is coated with a metal layer that acts as a current collector. The bottom electrode is first coated with a metal layer for the conduction and then with a dielectric layer (e.g. Al_2_O_3_ or SiO_2_) with an additional layer of fluoropolymer (e.g. Teflon or CYTOP) for surface hydrophobicity. An electrolyte is sandwiched between the electrodes which upon oscillation generates an AC current. The AC current generation in REWOD depends on several parameters such as dielectric material, surface charge density, surface hydrophobicity (to minimize contact angle hysteresis and liquid pinning effects), modulation frequency, and electrode–electrolyte interfacial area. These parameters directly or indirectly influence the capacitance in the dielectric material and therefore affect the AC current generation from the energy harvester.

Capacitance can be expressed as *C* = *ε*_0_*ε*_*r*_*A/d* where *ε*_0_ = 8.85 × 10^–12^ F/m is the vacuum permittivity, *ε*_*r*_ is the relative permittivity of the dielectric material, *A* is the electrode–electrolyte interfacial area, and *d* is the thickness of the dielectric layer. Two ways capacitance can be increased are by decreasing the dielectric thickness or by using dielectric materials with high *ε*_*r*_. Many high *ε*_*r*_ materials have been reported to have high resistivity and thermal instability^22^, which presents a limitation on dielectric materials that are ideal for REWOD energy harvesting. Another phenomenon affecting capacitance is the leakage current, which needs to be prevented or minimized to achieve a higher AC current. Comparisons between amorphous and crystalline thin films have been made to show that crystalline films have grain structure that contributes to a higher leakage current^23^. E-beam evaporation, sputtering, and atomic layer deposition (ALD) have been used to provide dense and uniform dielectric films and hence minimize the leakage current^24^.

Various theoretical modeling studies accompanied by experimental validation have been previously published, but the research thrust on optimizing the electrode–electrolyte combinations to maximize harvested energy has been rather limited. Moon et al*.* proposed a REWOD model using two dissimilar dielectrics with a significant difference in surface charge density^21^. Because this work did not use a bias voltage, the EDL capacitance was significant. Using plain water as an electrolyte, the dissimilar dielectrics produced different capacitances, which resulted in AC current. This was the first REWOD study that did not use any bias voltage and the authors reported a power density of 0.3 µW/cm^2^. Yu et al. followed a similar approach, thus producing different EDL capacitances at a given frequency by making alternating contacts between a water drop electrolyte and electrodes coated with PTFE and CYTOP as dielectrics^25^. Yang et al*.* used a thin film layered structure where a very high *ε*_*r*_ dielectric (TiO_2_) layer was blocked with a secondary layer of much lower *ε*_*r*_ dielectric (Al_2_O_3_) as a leakage barrier layer^26^. The layered structure significantly increased the REWOD capacitance while preventing the leakage current. This resulted in 15.36 mW/cm^2^ of power density with an applied bias voltage of 30 V.

All previous researches on REWOD energy harvesting successfully demonstrated AC current generation. It is, however, apparent that these works used limited parameter configurations and did not attempt to optimize various parameter combinations to maximize the power density. The present work leads to a novel path toward optimized electrode–electrolyte configurations, validated both experimentally and theoretically, to make REWOD a feasible source of power in wearable sensors. Additionally, because no external bias voltage was applied in this work, concerns over the breakdown strength of the dielectric are eliminated. Very high breakdown strength due to the “zero applied-bias-voltage” makes the proposed energy harvester possibly much more reliable and increases the device longevity.

Our recent work in implementing charge amplifier and DC–DC converter integration into the REWOD energy harvester can produce sufficient DC power at low frequency (< 3 Hz) to self-power wearable motion sensors^27^. In this work, a small input voltage from the REWOD is rectified to convert the AC signal to a DC voltage and then boosted and regulated to supply a constant DC power by using a DC–DC converter. The constant DC power is capable of powering the internal components of the integrated REWOD energy harvester; charge amplifier, analog-to-digital converter (ADC) that digitizes the amplified signal, and a transmitter (TX) that transmits the data wirelessly to a remote receiver to detect human motion as part of the self-powered motion sensor.

## Theoretical modeling

### Lumped element-based electrical circuit model

As discussed in the previous section, the electrode–electrolyte interface in REWOD forms a capacitance, which could be modeled as a parallel plate capacitor. As the capacitance increases with decreasing dielectric film thickness, the generated charge proportionally increases (*dQ* = *VdC*), where *dQ* and *dC* are the rate of change in the generated charge and capacitance, respectively, when the electrolyte is mechanically modulated. *V* is the inherent bias voltage across the electrodes due to the DC leakage current flowing through the transducer. For a given frequency, *f*, inherent bias voltage in the form of DC offset is a constant (shown in the Supplementary Fig. S5). During modulation, as the electrodes oscillate at an input frequency, *f*, an AC current is generated through the capacitor due to the continuous charging and discharging with respect to the change in the electrode–electrolyte interfacial area.

**Figure 2:**
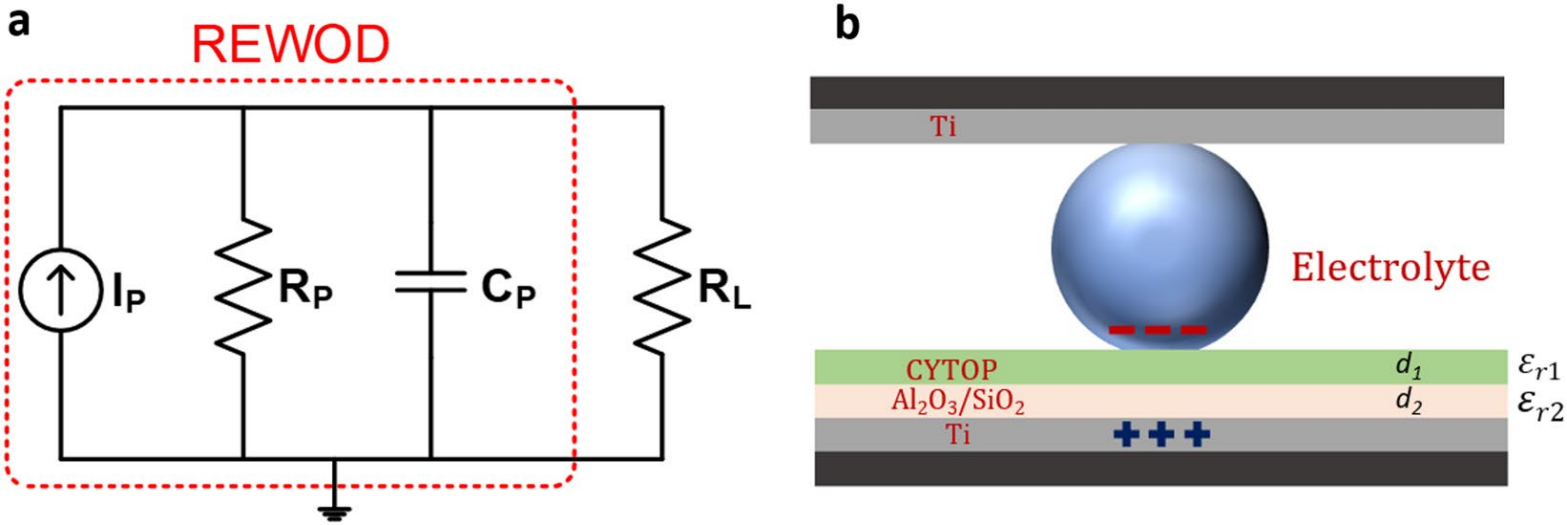
(**a**) Lumped element model of a REWOD energy harvester, (**b**) electrolyte bridge between dielectric and conductive layers showing relevant dielectric constants (*ε*_*r*1_ and *ε*_*r*2_) of the dielectric layers and their thicknesses (*d*_1_ and *d*_2_).

The AC current generation associated with the electrolyte modulation between the electrodes thus depends on the change in the capacitance between them. Theoretically, the REWOD can be approximately modeled using lumped element-based electrical circuit components as shown in Fig. 2a. As discussed in the introduction section, the electrical double layer capacitance (EDLC) that is formed at the electrolyte-metal (top electrode) interface is much larger than the capacitance formed at the electrolyte-dielectric (bottom electrode) interface. However, when the EDLC is added in series with the electrolyte-dielectric capacitance, it has a negligible contribution to the equivalent capacitance. Thus, we modeled the REWOD capacitance considering only the electrolyte-dielectric interface. The model includes a resistor *R*_*P*_, a capacitor *C*_*P*_, and a current source *I*_*P*_ in parallel. Parallel connection as opposed to series connection eliminates the complexity of requiring two different voltages across the resistor and capacitor in the modeling. Most piezoelectric and pyroelectric energy harvesting models assume parallel connection with current source^28,29^. *I*_*P*_ represents generated AC current which is the rate of change of generated charge across the REWOD electrodes. *C*_*P*_ acts as a variable capacitor that changes periodically during the electrolyte modulation, while electrical resistance, *R*_*P*_, occurs across the electrodes due to the electrical conductivity and thicknesses of the electrolyte, dielectric, and conductive layers along with electrode–electrolyte interfacial area. The effective capacitance, *C*_*P*_, and resistance, *R*_*P*_, of the model can be estimated using Eqs. (1) and (2) taking into consideration the thicknesses of the dielectric layers.

1 $$ C_{p} = \frac{{\upvarepsilon _{eff} A}}{{d_{1} + d_{2} }} $$

2 $$ R_{p} = \frac{\rho l}{A} $$

where *A* is the electrode–electrolyte interfacial area while electrolyte is being modulated, *d*_*1*_ and *d*_*2*_ are the thicknesses of hydrophobic layer (CYTOP) and dielectric layer (Al_2_O_3_/SiO_2_), respectively, *ρ* is the resistivity, and *l* is the electrolyte layer thicknesses (variable distance between the electrodes). *ɛ*_*eff*_ is the effective dielectric constant of the hydrophobic layer and dielectric layer, which can be calculated using Eq. (3)^30^.

3 $$\upvarepsilon _{eff} = \frac{{d_{1} + d_{2} }}{{\frac{{d_{1} }}{{\upvarepsilon _{r1} }} + \frac{{d_{2} }}{{\upvarepsilon _{r2} }}}} $$

where *ɛ*_*r*1_ and *ɛ*_*r*2_ are the relative dielectric constants of the hydrophobic layer (CYTOP) and dielectric layer (Al_2_O_3_/SiO_2)_, respectively. Considering bulk material properties, the dielectric constants used in the calculation of the effective capacitance are: *ɛ*_*r*1_ = 2.1, and *ɛ*_*r*2_ = 9.1 or 3.9 (Al_2_O_3_ or SiO_2_), respectively^31^. Graphical representation of dielectric thicknesses *d*_*1*_ and *d*_*2*_ for both CYTOP and Al_2_O_3_/SiO_2_ along with their respective dielectric constants (*ɛ*_*r*1_ and *ɛ*_*r*2_) are depicted in Fig. 2b.

For any given electrode–electrolyte combination during modulation, the effective capacitance varies due to the continuous variation in the electrode–electrolyte interfacial area and the corresponding charges. Therefore, the generated AC current can be estimated as:

4 $$ I_{p} = \frac{dQ}{{dt}} $$

where, *dQ,* the rate of change in the generated charge, can be approximated using Eq. (5)^29,32^.

5 $$ dQ = P_{Q} \left( {t, \varepsilon ,f} \right)\sigma_{S} \left( M \right)dA\left( {V,l} \right) $$

where, *P*_*Q*_ is the surface charge coefficient, $\sigma_{s}$ is the surface charge density, and *dA* is the change in the electrode–electrolyte interfacial area during modulation, which is a function of electrolyte volume, *V*, and electrode displacement, *l*. Equation (5) is based on an analogous equation for pyroelectric generated charge and has been slightly modified for the present work based on the assumptions that surface charge density and interfacial area are directly proportional to generated charge. Surface charge densities of the CYTOP and Al_2_O_3_/SiO_2_ electrodes in contact with different molar concentration electrolytes are approximately determined as: 0.083, 0.040, and 0.030 in C/m^2^ for 1.0 M, 0.5 M, and 0.1 M NaCl aqueous solutions, respectively^33^. During the electrolyte modulation, the distance between the top and the bottom electrodes varies between displacements *l*_1_ (maximum distance, minimum electrode–electrolyte interfacial area) and *l*_*2*_ (minimum distance, maximum electrode–electrolyte interfacial area), as shown in Fig. 1. Surface charge coefficient, *P*_*Q*_, is an empirically derived parameter associated with the dielectric thickness and permittivity of the dielectric-electrolyte layers. *P*_*Q*_ is also a function of the oscillating frequency, *f*, and is calculated from the measured charge over time considering the frequency and variable electrode–electrolyte interfacial area upon modulation of any specific electrolyte. *dQ* values are first determined using Eq. (5) for both the measurement results and the theoretical model, which are subsequently matched with one another using the *P*_*Q*_ values. For instance, in the case of an electrode with a 50 nm CYTOP layer, 100 nm SiO_2_ layer, and 1.0 M NaCl electrolyte at 1 Hz modulation frequency, value of *P*_*Q*_ was empirically determined to be 0.007. *P*_*Q*_ values for all the combinations of electrodes, electrolytes, and frequencies are determined and used in theoretical current density calculations. For demonstration purposes, *P*_*Q*_ values for a combination of 1 Hz modulation frequency with 1.0 M NaCl solution as an electrolyte for all the dielectric thicknesses of each dielectric samples are presented in Table 1.

**Table 1:** Representative measured empirical surface charge coefficients for three different thicknesses of Al_2_O_3_ and SiO_2_ as dielectrics (100 nm, 150 nm, and 200 nm) with 1.0 M NaCl electrolyte and 1 Hz input frequency.

Dielectric thickness (nm)	*P*_*Q*_ (Al_2_O_3_)	*P*_*Q*_ (SiO_2_)
100	0.009	0.007
150	0.007	0.006
200	0.0055	0.005

## Experimental section

### Electrode fabrication and electrolyte preparation

Two dissimilar electrodes were fabricated using (single side polished) highly doped P-type silicon wafers with a diameter of 50.5 mm and a thickness of 0.38 mm (University Wafers Inc). Electrodes are referred to as dissimilar if they are either coated with different dielectrics or one is coated with dielectric and another with metal. Both wafers were first coated with a ~ 100-nm-thick titanium adhesion layer. Before the dielectric material deposition over the titanium layer, a small portion of the wafer was covered with Kapton tape to block the dielectric insulation and later removed to enable current conduction. Three different thicknesses (100 nm, 150 nm, and 200 nm) of dielectric materials (Al_2_O_3_ and SiO_2_) were separately deposited, making six samples, each with a separate dielectric material and thicknesses. The titanium and dielectric materials were deposited using a NEE-400 dual e-beam evaporator (Nanomaster Inc). After each deposition, thicknesses were verified using the Alpha-Step D-300 Stylus Profiler (KLA Corporation).

**Figure 3:**
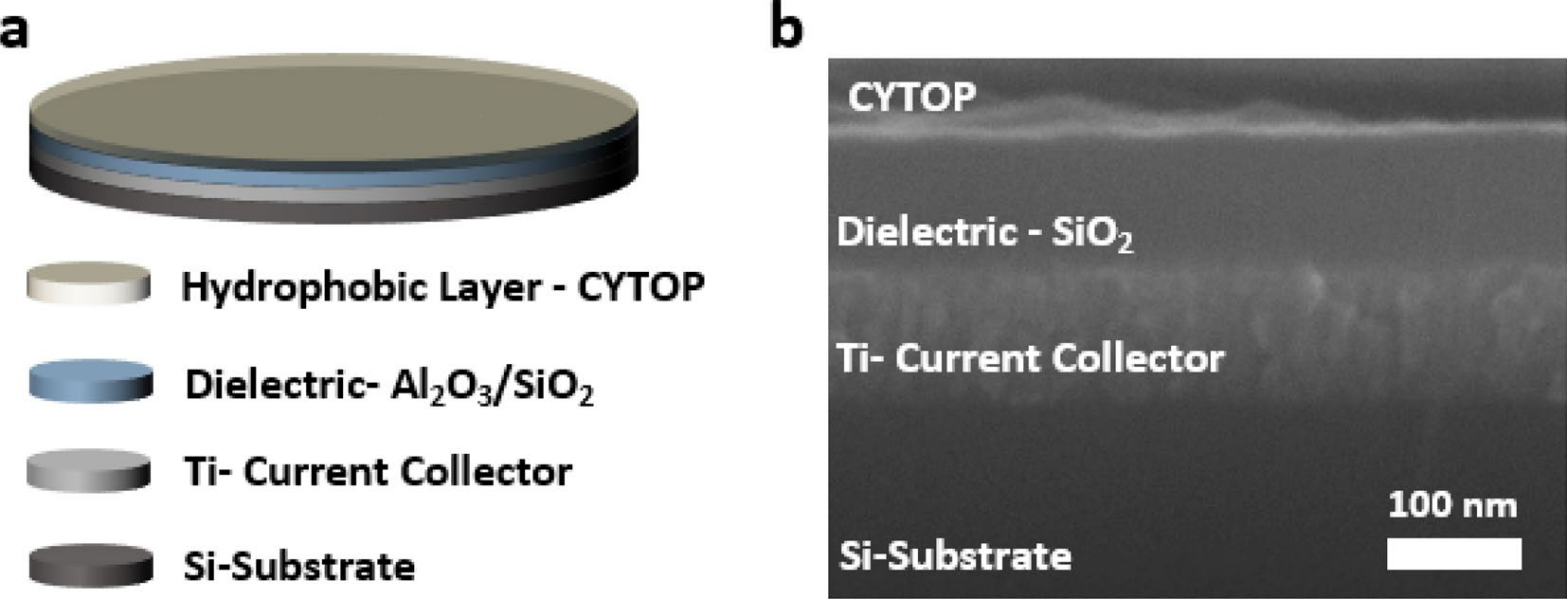
(**a**) Schematic of the metal and dielectric electrodes used for REWOD energy harvesting. (**b**) A cross-sectional view of a fabricated sample obtained by FE-SEM. The thickness of the titanium electrode was about 100 nm, and that of the Al_2_O_3_/SiO_2_ dielectric layer was either 100 nm, 150 nm, or 200 nm (100 nm SiO_2_ chosen for the SEM image).

After a successful deposition of the desired thickness of Al_2_O_3_ and SiO_2_, the wafers were deposited with an additional layer of hydrophobic material. A fluoropolymer, CYTOP (CTL-809 M), and its solvent (CT-Solv. 180), both purchased from AGC Chemicals Company, were mixed together in a volumetric ratio of 1:3. The solution was spin-coated on the wafers over the dielectric layer. Spin coating was performed at 600 rpm for 5 s (spread cycle) and then 3000 rpm for 50 s (spin cycle). The samples were dried at room temperature for 15 min, pre-baked for 30 min at 80 °C, and final-baked for 60 min at 185 °C to ensure complete evaporation of the solvent. A complete sample is illustrated in Fig. 3a showing the layered electrode structure. An SEM image of the cross-section was obtained using field-emission scanning electron microscopy (JEOL JSM-7001F), as shown in Fig. 3b, to confirm the thickness and uniformity of different deposited layers.

Sodium chloride (Sigma Aldrich Inc.) aqueous solutions of 0.1 M, 0.5 M, and 1.0 M concentrations and 20 mL each were prepared in deionized water and used as electrolytes. Although such aqueous solutions are not ideal in proposed REWOD applications due to their volatility, they were chosen to facilitate the study on how changing molar concentration, and hence the variation in the surface charge density affects the current/power output.

### Contact surface area calculation

**Figure 4:**
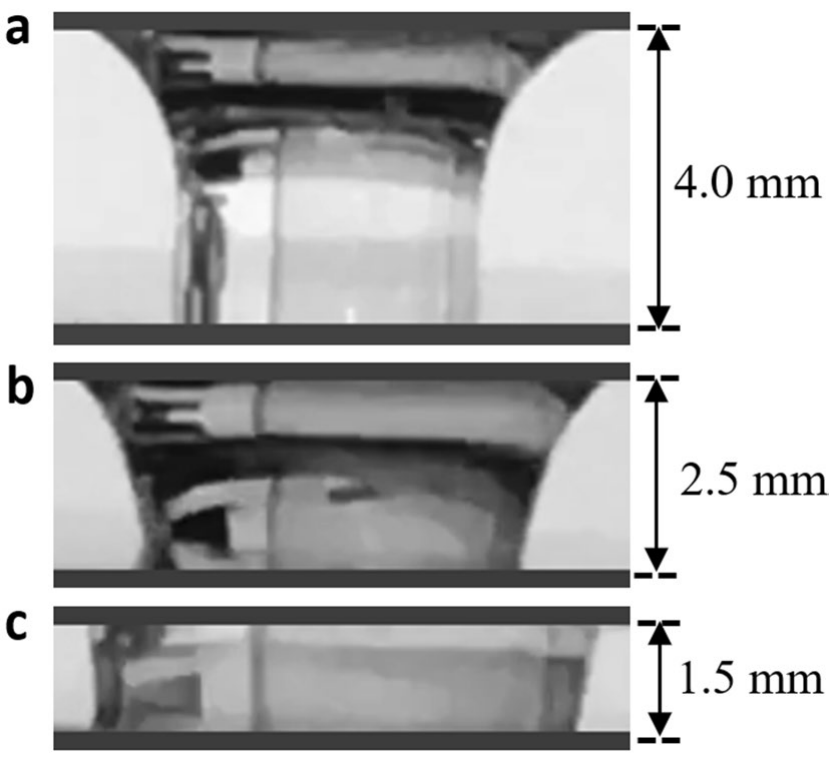
Video freeze shots of three stages of electrolyte between electrodes during modulation: (**a**) 4 mm apart, (**b**) ~ 2.5 mm apart, and (**c**) 1.5 mm apart. Electrolyte geometry suggests that the liquid completely forms a cylindrical disk while electrodes are at the minimum distance apart (maximum electrode–electrolyte interfacial area).

When an electrolyte of volume *V* is modulated between the two electrodes, the shape of the liquid changes periodically. Based on the change in the electrolyte shape observed during the modulation, a geometrical approach was adopted to best estimate the electrode–electrolyte interfacial area. As shown in Fig. 4a, at the maximum displacement (minimum electrode–electrolyte interfacial area) of *l*_*1*_ = 4 mm, the top electrode (without a dielectric layer) shows higher affinity to the electrolyte compared to that of the bottom electrode (with a dielectric-hydrophobic layer) resulting in a slightly higher electrode–electrolyte interfacial area with the top electrode (*A*_*T*_) than that with the bottom electrode (*A*_*B*_).

According to Young’s equation; $\gamma_{sl} = \gamma_{sg} - \gamma_{\lg } cos\theta$; where *s*, *l*, and *g* represent solid, liquid, and gas, respectively; *γ* is the surface energy between the two media, and *θ* is the contact angle between the solid- liquid interface. The CYTOP coating (hydrophobic) makes a much larger contact angle with liquid compared to the hydrophilic surface (titanium). Referring to the Young’s equation, since $\gamma_{sg} \;{\text{and}}\;\gamma_{lg}$ are constants, the value of $cos\theta$ decreases for higher contact angles and hence the surface energy ($\gamma_{sl} )$ term also decreases for higher contact angles. Therefore, there is a much stronger attraction between the Ti-liquid interface compared to the CYTOP-liquid interface implying that the interfacial area between CYTOP-liquid periodically changes while that between Ti-liquid remains relatively constant.

As the modulation begins, transitioning from Fig. 4a,b, the electrode–electrolyte interfacial area at the top electrode stays relatively constant while it increases for the bottom electrode. At the minimum displacement (maximum electrode–electrolyte interfacial area) of *l*_*2*_ = 1.5 mm (Fig. 4c), the electrolyte forms an almost uniform cylindrical disk (*A*_*T*_* ≈ A*_*B*_). The volume of the cylindrical-shaped liquid droplet can be approximated as:

6 $$ V = \pi r^{2} l $$

where *r* is the radius of the cylindrical disk and *l* is the distance between the two electrodes at the minimum displacement (*l*_*2*_ = 1.5 mm). The electrode–electrolyte interfacial area was calculated using the known volume of the droplet, the distance between the electrodes, and the equation for the volume of a cylindrical disk as given in Eq. (6). For a 50 µL electrolyte droplet, the maximum electrode–electrolyte interfacial area (*πr*^2^) was calculated to be approximately 0.33 cm^2^.

### Measurement set-up and experiments

**Figure 5:**
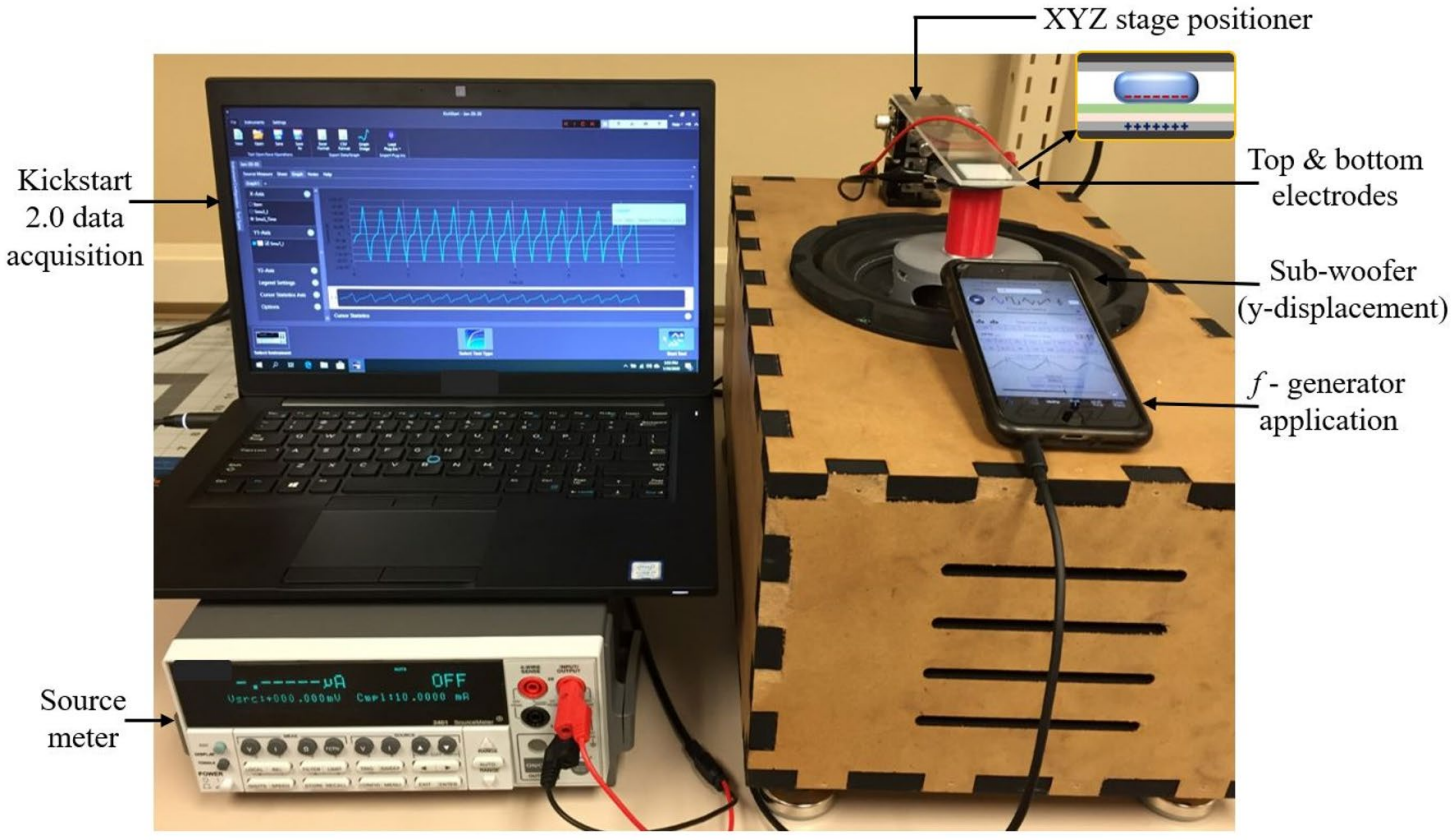
Measurement set-up for REWOD energy harvesting. Electrolyte modulation between the two dissimilar electrodes is shown in the inset.

The measurement set-up for the AC current and power generation measurement is illustrated in Fig. 5. It consisted of an XYZ positioner stage with a long and lightweight acrylic beam attached to it. The beam was used to hold the top electrode stationary. The y-axis of the XYZ positioner was set to a distance of 4 mm from the bottom electrode before the modulation started. Input oscillations were applied using a custom-built subwoofer system that could be controlled with a signal generating app (Audio Function Generator PRO). This application works almost the same way as the actual function generator, except it excites the subwoofer in vertical mechanical displacement to a desired amplitude. The shaker system consisted of an 8-inch 800-W subwoofer (Pyle), a 400 W amplifier (Boss CX250), and a 12-V power source (Apevia ATX Raptor) attached to a power adapter cord. Similar custom-made systems have been reported in prior energy harvesting research^34–36^. This was a simple, inexpensive method for generating low-frequency and relatively high amplitude oscillations. A custom wood enclosure provided a location to mount the subwoofer and also contained the amplifier and power source. A 3D-printed sample-holding stage was placed over the subwoofer dust cap to provide a flat surface to hold the bottom REWOD substrate. During the electrolyte modulation between the electrodes, the generated AC current was measured using a Keithley 2400 Sourcemeter and the measurement results were acquired using the Keithley data acquisition software, Kickstart 2.0.

The displacement amplitude for each subwoofer frequency was determined with the corresponding vertical distance measurements during the modulation using a slow-motion camera and a ruler. The input oscillation frequencies were applied in the range of 1–3 Hz with 0.25 Hz step using the function generator application (Audio Function Generator PRO). The amplitudes for all the frequencies were adjusted in the application for the bottom electrode to be displaced vertically from an initial gap of 4 mm from the top electrode to 1.5 mm from the top electrode, resulting in a net vertical displacement of 2.5 mm. A representative wave form of the excitation at 1 Hz frequency showing the measured electrode gap with respect to time is shown in Fig. S6.

## Results and discussion

In order to maximize the output generated power in REWOD energy harvesting, a high-quality dielectric thin film is crucial. There are several ways to verify the quality of dielectric thin films as well as the surface hydrophobicity. Low leakage current between the electrodes when the electrolyte is at rest ensures a high-quality thin film as it indicates there are very few pathways in the thin film. A voltage difference across the capacitor is required to drive the current through the REWOD. In the absence of bias voltage, a small inherent voltage that is present across the capacitor drives the leakage current. Leakage current, in the absence of the bias voltage and without substrate oscillation was measured for all the samples and found to be within the 1.2–1.8 nA/cm^2^ range. This shows that compared to the total current density output, leakage current is insignificant. The leakage current that occurs in the REWOD energy harvester is because of the fact that the dielectric layer is not a perfect insulator and can be considered a loss term^24^.

**Figure 6:**
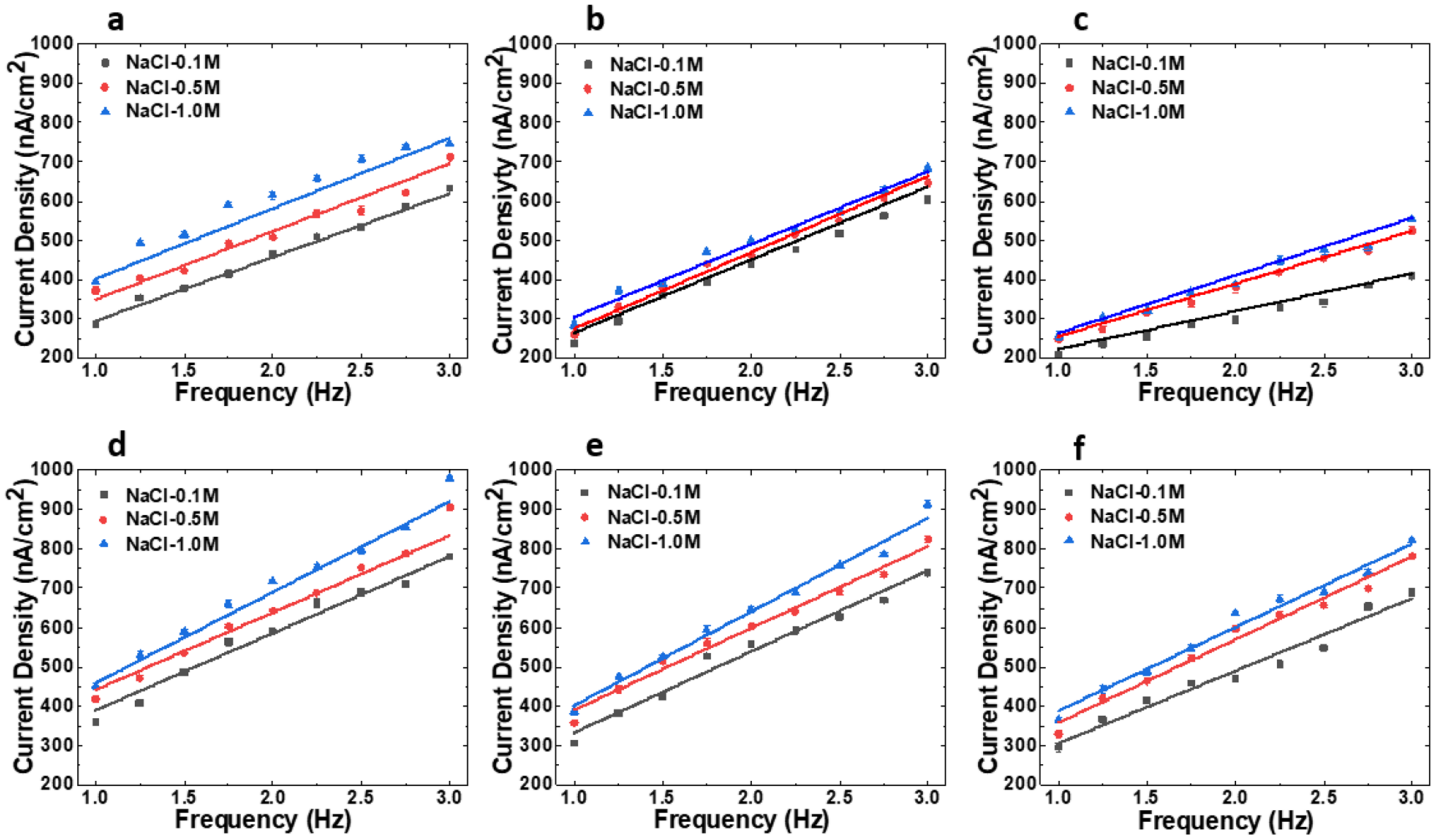
Current density (nA/cm^2^) plotted against frequencies (Hz) for (**a**) SiO_2_-100 nm, (**b**) SiO_2_-150 nm, (**c**) SiO_2_-200 nm, (**d**) Al_2_O_3_-100 nm, (**e**) Al_2_O_3_-150 nm, and (**f**) Al_2_O_3_-200 nm. Corresponding linear-fit solid lines represent data from the theoretical model.

Figure 6 shows the current density for nine different frequencies (1–3 Hz with a 0.25 Hz step size) for all the combinations of the dielectrics and the electrolytes used in this work. Each measurement was repeated three times and the average result is presented with an error bar representing + /− one standard deviation from the mean. The corresponding current densities obtained from theoretical modeling were plotted as straight-line linear fits showing that the results from the theoretical model align well with the measured current densities. One of the reasons why the theoretical model fits the measurement results very well is that the measured data were used to obtain *P*_*Q*_ in Eq. (5) as discussed in the modeling section. The results follow a linear pattern where an increasing frequency increases the output current. Current density increased with a higher dielectric constant, thinner dielectric coating, and higher surface charge density. As expected, the highest output current density of ~ 1 µA/cm^2^ was measured using the combination of 100 nm Al_2_O_3_ and 1.0 M NaCl electrolyte at 3 Hz frequency. This magnitude of current is significant given the fact that it completely arose within the electrode–electrolyte interface without any external bias voltage. The modulation frequency range used in this work falls well within the range of a person’s walking or running frequency, which illustrates that the energy harvester can efficiently operate in such motion frequencies. Another observation from the results depicted in Fig. 6 is that a higher NaCl molarity resulted in a higher current density, presumably because of the increased surface charge density and therefore higher capacitance.

**Figure 7:**
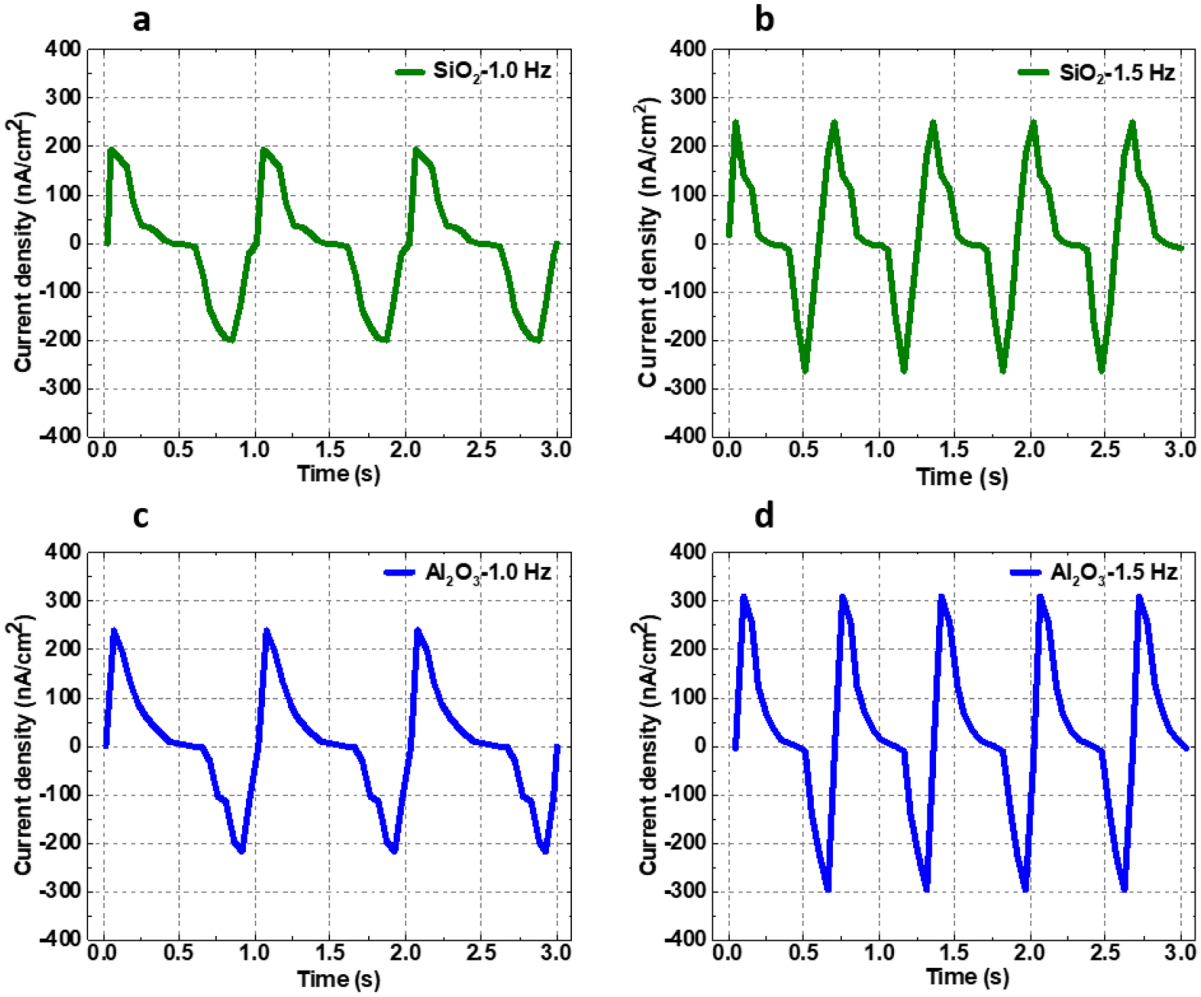
Representative AC peak-to-peak current density plots for the first three seconds of mechanical oscillation and using 1.0 M NaCl electrolyte for the following: (**a**) SiO_2_, 100 nm, 1 Hz; (**b**) SiO_2_, 100 nm, 1.5 Hz; (**c**) Al_2_O_3_, 100 nm, 1 Hz; and (**d**) Al_2_O_3_, 100 nm, 1.5 Hz. The positive and negative peaks are slightly different indicating charging and discharging characteristics of the capacitor.

Figure 7 provides a few representative samples of measured current density vs. time over a 3-s time period for selected electrode–electrolyte configurations and at two different frequencies (1 Hz and 1.5 Hz). Additional representative measured AC signal plots are provided in Supplementary Figs. S1–S4. The AC signal for each of the measurements shows very little differences in the magnitude and shape of the charge/discharge curves, indicating insignificant contact angle hysteresis and liquid pinning, which can limit droplet movement (and therefore peak-to-peak current). From the figures, it is also observed that the difference between the charging and the discharging time, the time constant (τ), are different for different frequencies agreeing with the theoretical relationship between the time constant and the frequency (*τ* = *R*_*P*_*C*_*P*_ = *1/2πf*). For instance, the time constants for Al_2_O_3_ at 1 Hz and 1.5 Hz frequencies are approximately 0.6 s and 0.45 s, respectively. Among the representative AC peak-to peak current density plots, there is a range of variation between positive and negative peaks. Spatial variation in trapped charges within the dielectric layers could create DC bias. This DC bias also causes the positive and negative peak differ in magnitude that can vary from electrode to electrode.

As it can be observed in Fig. 7, the AC signals are not sinusoidal, which could be explained as follows: The contact surface area between the electrode and electrolyte has a key role in generating the AC current. Since the wetting properties for the two electrode surfaces are different, the contact surface area does not remain the same for the charging and discharging period during the oscillation. As the electrolyte contact area at the top electrode changes at a different rate than that at the bottom electrode, two different *RC* values for charging and discharging occur. Since the change in the surface area is not purely sinusoidal, this results in the nonlinearity of the varying capacitance ($C = \varepsilon A{/}d$). Hence, the generated AC current does not follow an ideal sinusoidal waveform.

**Figure 8:**
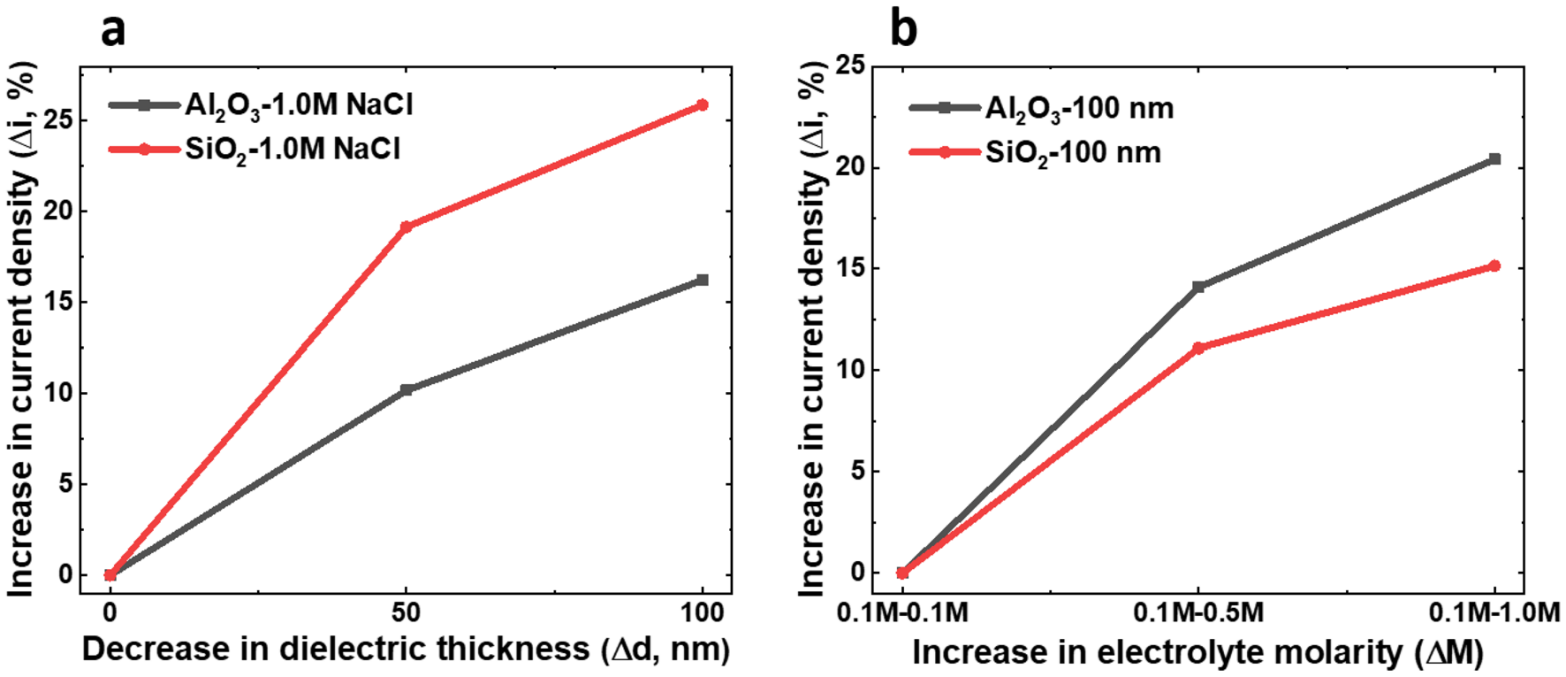
Representative changes (percent increase in current density) with (**a**) decrease in dielectric film thickness (nm) for Al_2_O_3_ and SiO_2_ dielectrics with 1.0 M NaCl electrolyte and (**b**) increase in electrolyte molarity for 100 nm Al_2_O_3_ and 100 nm SiO_2_ dielectrics, at 3 Hz input frequency.

Among various factors contributing to the maximum power output in REWOD, some factors have greater influence than others. In order to compare how current density changes for the various electrode–electrolyte combinations, the percentage current density increase as a function of a decrease in the dielectric thickness and increase in electrolyte molarity were analyzed. Figure 8a shows how current density increases in percentage (*Δi* %) with decreasing dielectric thickness for both Al_2_O_3_ and SiO_2_ thin film samples with 1.0 M NaCl electrolyte at 3 Hz input frequency. From the results, it is observed that for both Al_2_O_3_ and SiO_2_ reduced thin films have a significant influence on current density. However, reduction in SiO_2_ thickness had a greater influence on current density than that of Al_2_O_3_ (~ 25% current density increase for SiO_2_ vs ~ 15 percent increase for Al_2_O_3_ with 100 nm dielectric thickness reduction). As discussed previously, dielectric materials with lower dielectric constants have lower resistivity than those with higher dielectric constants. Therefore, a higher percentage current density increase is observed with decreasing SiO_2_ film thickness compared to the Al_2_O_3_ film thickness. Over twenty-five percent increase in current density is observed when SiO_2_ film thickness was reduced to 100 nm from 200 nm. Since there is no restriction to breakdown voltage, dielectric thickness could be reduced to just a few tens of nanometer, thereby increasing the current density by over fifty percent, assuming the proportionality holds. To put it into perspective, the breakdown strength of Al_2_O_3_ thin film dielectrics deposited with e-beam evaporation has been shown to be ~ 0.5–0.8 V/nm^37^, implying that if even just a few mV of DC offset were applied, less than 1 nm of Al_2_O_3_ would be needed for a REWOD energy harvester. By using physical vapor deposition (PVD) systems such as E-beam evaporation and sputtering, and atomic layer deposition (ALD), conformal and uniform deposition thickness could be very well controlled to a few tens of nanometer. Al_2_O_3_ and SiO_2_ films have been deposited in single-digit nanometer thicknesses successfully using ALD^37,38^. However, there will certainly be challenges to create uniform coatings of single digit to a few tens of nanometers thickness due to coating nucleation and migration at the surface. In addition, the coatings obviously become more susceptible to mechanical damages as they become very thin.

Additionally, the percentage increase in current density as a function of increasing electrolyte molarity for 100 nm samples of both Al_2_O_3_ and SiO_2_, also at 3 Hz input frequency, is shown in Fig. 8b. An increase in electrolyte molarity showed a less significant percentage increase in current density compared to that of the decreasing dielectric film thickness. Whereas SiO_2_ thin films showed a higher percentage current density increase with decreasing dielectric thickness, Al_2_O_3_ thin films showed a higher percentage current density increase with increasing electrolyte molarity.

In reiteration, this study mainly focused on how decreasing dielectric thickness with varying electrolytes enhanced REWOD current generation. However, an alternative optimization approach is to use CYTOP as an electret and to investigate optimal CYTOP pre-charging. An electret is a dielectric with quasi-permanent charges on its surface. The charges trapped in proper electret materials are able to generate an electrostatic field for tens of years^39^. CYTOP is an well-known electret and even without using corona discharge, the inherent voltage of the CYTOP might be enhanced through the use of charge injection during electrowetting. CYTOP as a pre-charged electret has been successfully used in other energy harvesting technologies^40^.

Resistances and capacitances of all dielectric film thicknesses for both Al_2_O_3_ and SiO_2_ were measured using the Keithley 2400 Sourcemeter. The measured and theoretical capacitance (*C*_*P*_) and measured resistances (*R*_*P*_) of three different dielectric thicknesses of Al_2_O_3_ and SiO_2_ with 1.0 M NaCl electrolyte at the maximum electrode–electrolyte interfacial area are reported in Table 2. As expected from the theoretical modeling, the capacitance exhibited an inverse relationship with the thickness of the dielectric layer.

**Table 2:** Measured and theoretical capacitance (*C*_*P*_) and measured resistances (*R*_*P*_) of three different dielectric thicknesses of Al_2_O_3_ and SiO_2_ with 1.0 M NaCl electrolyte at maximum electrode–electrolyte interfacial area.

Dielectric thickness (nm)	SiO_2_ (*C*_*P*_ in µF)		Al_2_O_3_ (*C*_*P*_ in µF)		Measured *R*_*P*_ (MΩ)	
	Measured	Theoretical	Measured	Theoretical	SiO_2_	Al_2_O_3_
100	0.312	0.3	0.351	0.381	2.20	2.05
150	0.267	0.261	0.276	0.3	1.80	2.05
200	0.21	0.222	0.255	0.237	1.70	1.90

Resistance is minimum when the electrodes are closest together at which point the capacitance is maximum due to the largest electrode–electrolyte interfacial area. Using the resistance and capacitance at optimum (minimum electrode gap), the equivalent impedance (*|Z|*) of the REWOD is determined using Eq. (7).

7 $$ \left| Z \right| = \frac{1}{{\sqrt {\left( {\frac{1}{{R_{P} }}} \right)^{2} + \left( {\omega C_{P} } \right)^{2} } }} $$

where *R*_*P*_ and *C*_*P*_ (in parallel) are the resistance and capacitance respectively, *ω* is the radian frequency, which is equal to *2πf,* and *f* is the modulation frequency in Hz. For 100 nm-Al_2_O_3_ sample at 3 Hz modulation frequency, using *R*_*P*_ and *C*_*P*_ from Table 2, the equivalent impedance *|Z|* using Eq. (7) is calculated to be 0.15 MΩ. The maximum power can be harvested when equivalent impedance (*|Z|*) is matched with an optimal external load. Considering an optimal load such that *|Z|* = |*Z*_*L*_|, the root mean square (RMS) power density is calculated using Eq. (8).

8 $$ P = \frac{{V_{RMS}^{2} }}{{4Z_{L} A}} $$

where *V*_*RMS*_ is the RMS voltage, *Z*_*L*_ is an optimal load, and *A* is the electrode–electrolyte interfacial area. Using RMS voltage of 0.103 V for 100 nm-Al_2_O_3_ electrode at 3 Hz modulation frequency (Fig. S5), the power with the optimal load is calculated to be 17.6 nW which corresponds to 53.3 nW/cm^2^ of true power density. In open circuit condition (without load), the power density increases four times to 0.21 µW/cm^2^. Power density from this work is fairly low, but scalable based on the proposed configuration. Considering the absence of bias voltage and the proposed avenue to enhance the current generation, bias-free REWOD energy harvesting could lead to fully self -powered wearable motion sensors.

In order to emphasize the significance of an energy harvester free of bias source, evaluation of the power density and other performance parameters of this work were compared to the other state-of-the-art works based on REWOD energy harvesting. We adopted and calculated a figure-of-merit (FOM) first proposed by Bassett et al.^41^ and is presented in Table 3. The FOM is defined as shown in Eq. (9).

9 $$ FOM = \frac{P}{{V^{2} fA}} $$

where *P* is the power, *V* is the applied bias voltage, *f* is the input oscillation frequency, and *A* is the electrode–electrolyte interfacial area. In order to calculate FOM for this work, it was necessary to measure the inherent voltage across the harvester due to the leakage current of the system as there was no applied bias voltage used in this work.

**Table 3:** Figure-of-merit (FOM) in logarithmic scale of various REWOD energy harvesting technologies demonstrated in the past and this work.

References	Output power (µW)	Area (mm^2^)	Bias voltage (V)	Frequency (Hz)	log_10_ [FOM (10^6^ µW/mm^2^ Hz V^2^)]
Huynh et al.^42^	0.192	200.00	1.2	6	2.05
Krupenkin et al.^18^	100	1.00	60	2	4.14
Hsu et al.^43^	80	0.80	4.5	300	4.22
Yang et al.^24^	137	1.25	24	3	4.80
This work	0.0533	100.00	0.01	3	6.25

Due to the leakage current, as discussed earlier, a small voltage is always present across the electrolyte in the form of DC offset. A 10 mV DC voltage was measured for Al_2_O_3_-100 nm-1.0 M NaCl-3 Hz configuration using an oscilloscope (Keysight Infinnivision DSOX3014A) and was used as the inherent DC bias to evaluate the power density of this work in terms of the FOM (Fig. S5). Most prior works reported either very low power densities or high bias voltages, which either way resulted in lower FOM compared to our work. Primarily, the higher FOM of this work can be credited to the “zero applied-bias-voltage” approach. FOM from this work was calculated to be three folds higher than the previously reported highest FOM. Therefore, the FOM was modified to a number in a logarithmic scale for comparison.

## Conclusion

In this work, REWOD energy harvesting technology was explored to optimize the electrode–electrolyte configuration and maximize the power output with the objective of eventually developing fully self-powered wearable sensors. Without an external bias source, AC current was generated, and the measurement results were verified with a theoretical model. Electrode–electrolyte configurations were varied using two different dielectric materials, three dielectric thicknesses, and three different electrolyte (NaCl) concentrations. 100 nm Al_2_O_3_ as dielectric and 1.0 M NaCl electrolyte at 3 Hz oscillation frequency, which was the highest frequency of all modulations, generated a current density close to 1.0 µA/cm^2^. A lumped element-based circuit model in which the electrolyte-dielectric configuration is in parallel was used to theoretically calculate current densities and verify the agreement with the experimental results. In order to estimate the power density, RMS AC voltage at 3 Hz frequency with 1.0 M NaCl electrolyte and 100 nm Al_2_O_3_ electrode was used with an optimal external load of 0.15 MΩ. At optimum, the power density of 53.3 nW/cm^2^ was calculated. Since no bias voltage was applied, the measured DC offset was used to calculate the FOM, which demonstrated a significant progress in REWOD energy harvesting related research works. This work also illustrated that REWOD has the potential for application to fully self-power wearable sensors without the need for an external bias source. Outcomes from this work fundamentally verified that the bias source-free energy harvesters are possible using REWOD technology. In addition, because there is no fear of voltage breakdown, dielectric thin films thickness could be further reduced to a much lower thickness to increase the power density.

## Supplementary Information

Supplementary Information.
