# Supplementary material for: Electrode and electrolyte configurations for low frequency motion energy harvesting based on reverse electrowetting
Source: Sci Rep. 2021 Mar 3;11:5030. doi: 10.1038/s41598-021-84414-3 (PMC7930057; doi:10.1038/s41598-021-84414-3)
Supplement: Supplementary file 1 — Supplementary Information. [file 41598_2021_84414_MOESM1_ESM.docx]

Supplementary Information

**Electrode and Electrolyte Configurations for Low Frequency Motion Energy Harvesting Based on Reverse Electrowetting**

Pashupati R. Adhikari^1,^ *, Nishat T. Tasneem^2^, Russell C. Reid^3^, Ifana Mahbub^2^

**^1^**University of North Texas, Department of Mechanical and Energy Engineering, Denton, Texas 76207

**^2^**University of North Texas, Department of Electrical Engineering, Denton, Texas 76207

^3^Dixie State University, Department of Engineering, St George, Utah 84770

***** Corresponding author. Department of Mechanical and Energy Engineering, University of North Texas, Denton, TX 76207

Email address: [pashupatiadhikari@my.unt.edu](mailto:pashupatiadhikari@my.unt.edu)


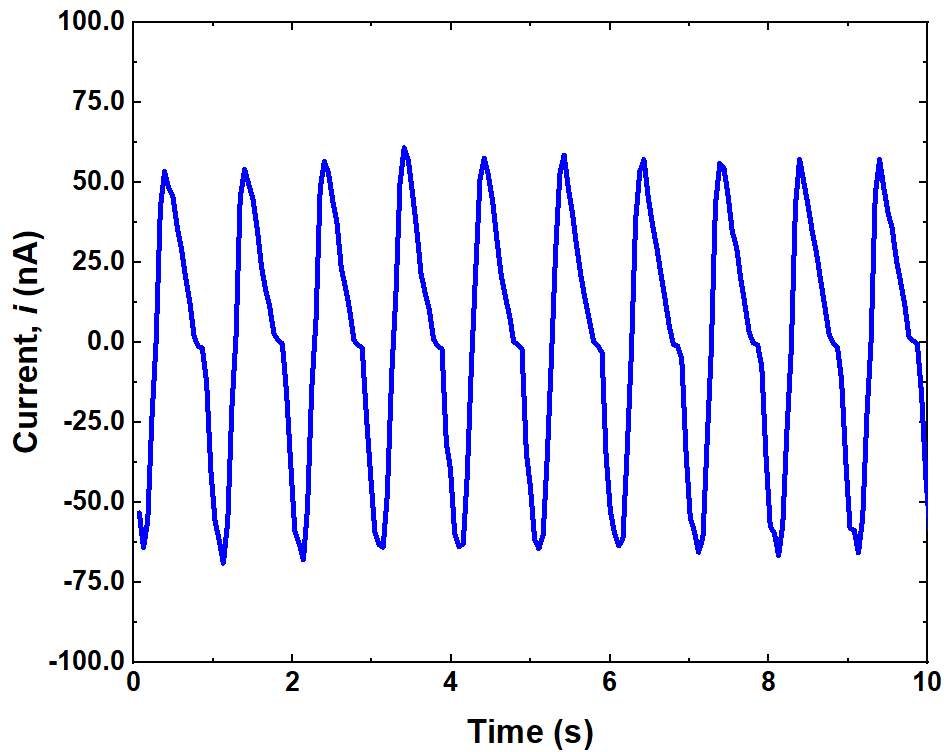


**Figure S1.** AC current generation (*i*) for Al_2_O_3_-150 nm-1.0M NaCl at 1.0 Hz frequency.


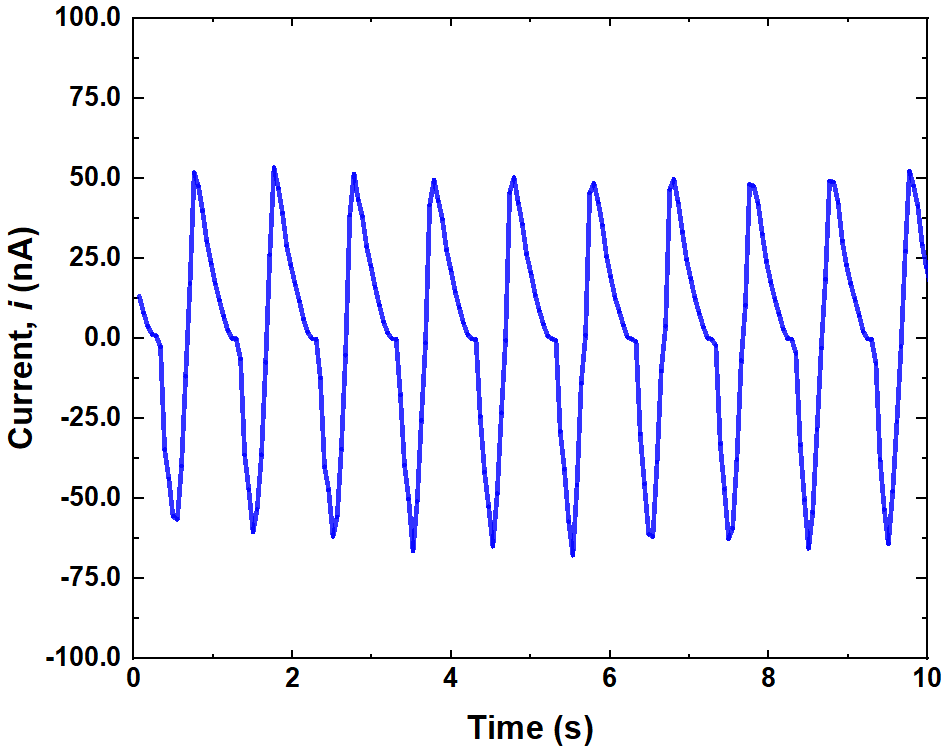


**Figure S2.** AC current generation (*i*) for Al_2_O_3_-200 nm-1.0M NaCl at 1.0 Hz frequency.


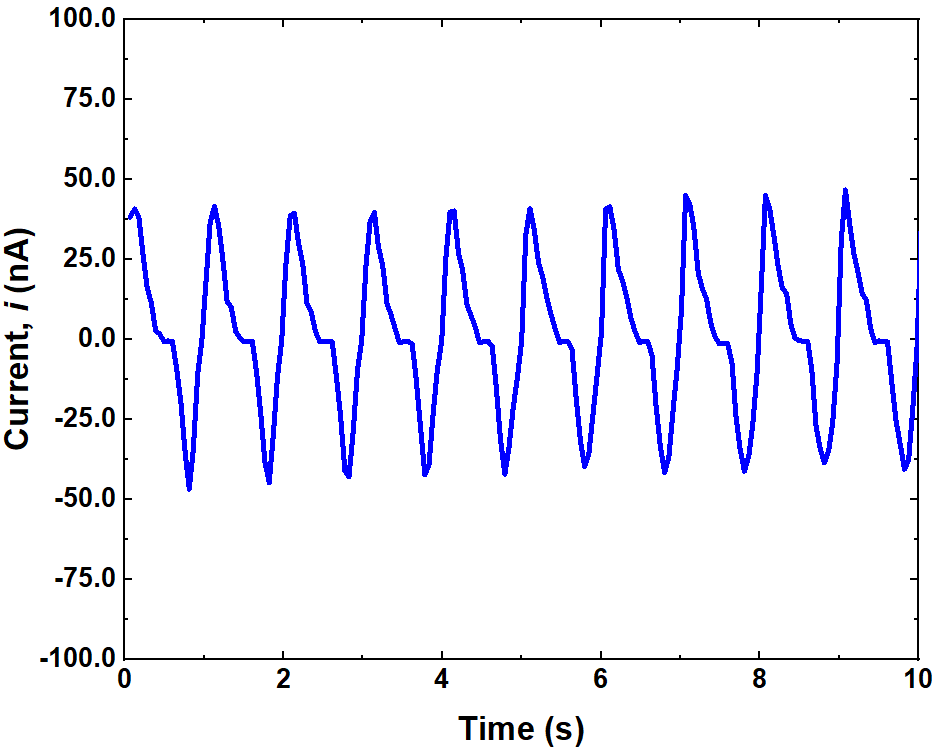


**Figure S3.** AC current generation (*i*) for SiO_2_-150 nm-1.0M NaCl at 1.0 Hz frequency.


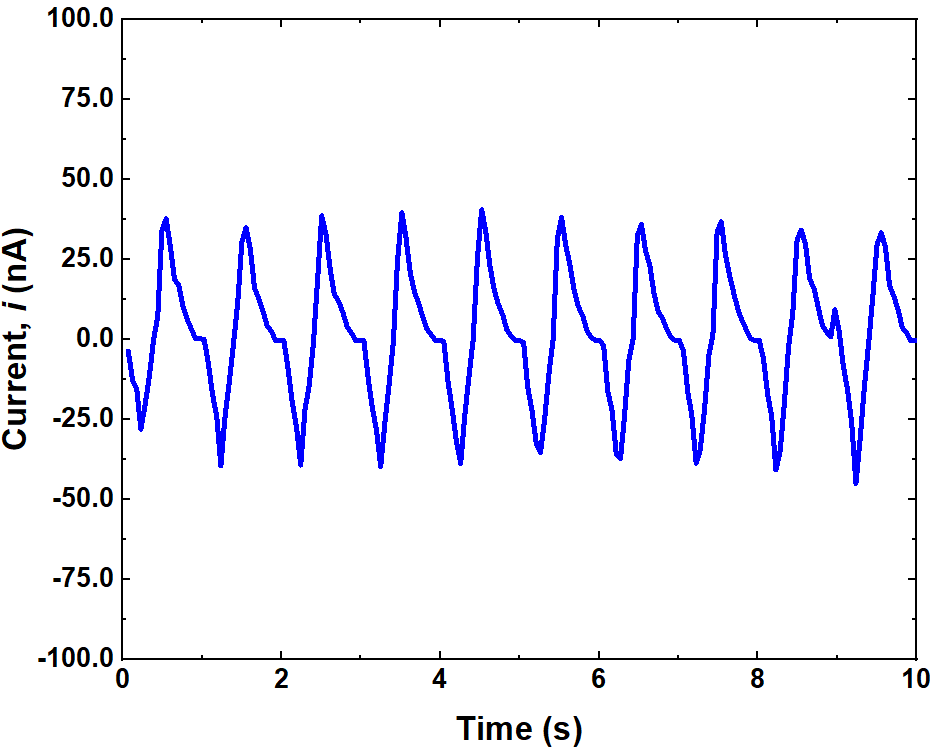


**Figure S4.** AC current generation (*i*) for SiO_2_-200 nm-1.0M NaCl at 1.0 Hz frequency.


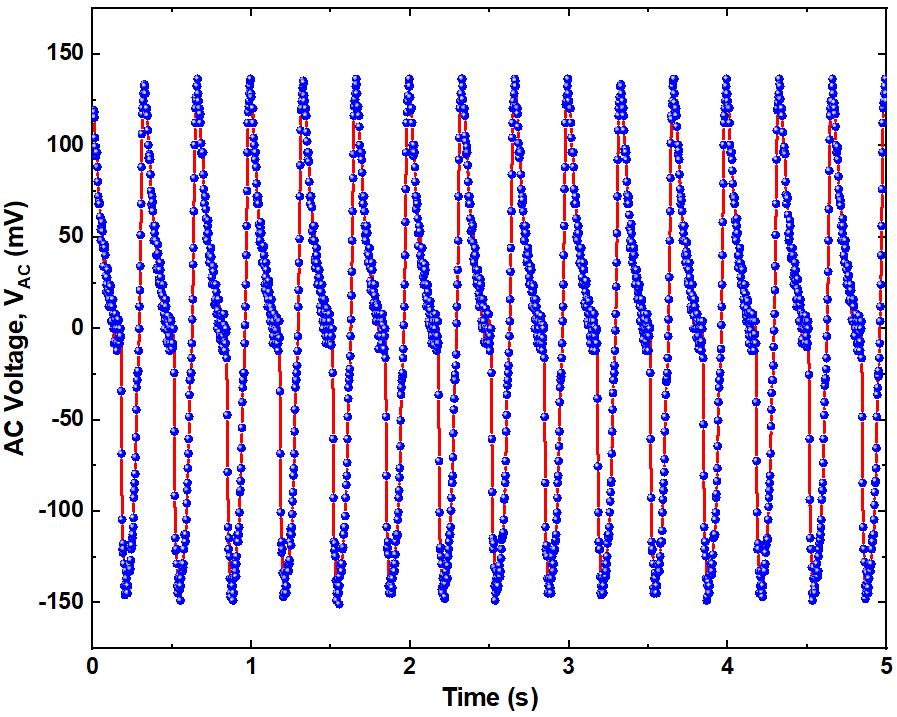


**Figure S5.** AC voltage (*V_AC_*) during modulation of Al_2_O_3_-100 nm-1.0 M NaCl configuration at 3.0 Hz frequency. The DC offset of ~10 mV, that is the difference between maximum positive peak of ~141 mV and maximum negative peak of ~ -151 mV is observed. During modulation, as the capacitance increases with decreasing dielectric film thickness, the generated charge proportionally increases (*dQ = VdC*), where *dQ* and *dC* are the changes in the generated charge and capacitance in REWOD respectively when the electrolyte is mechanically modulated, *V* is the inherent bias voltage across the electrodes.


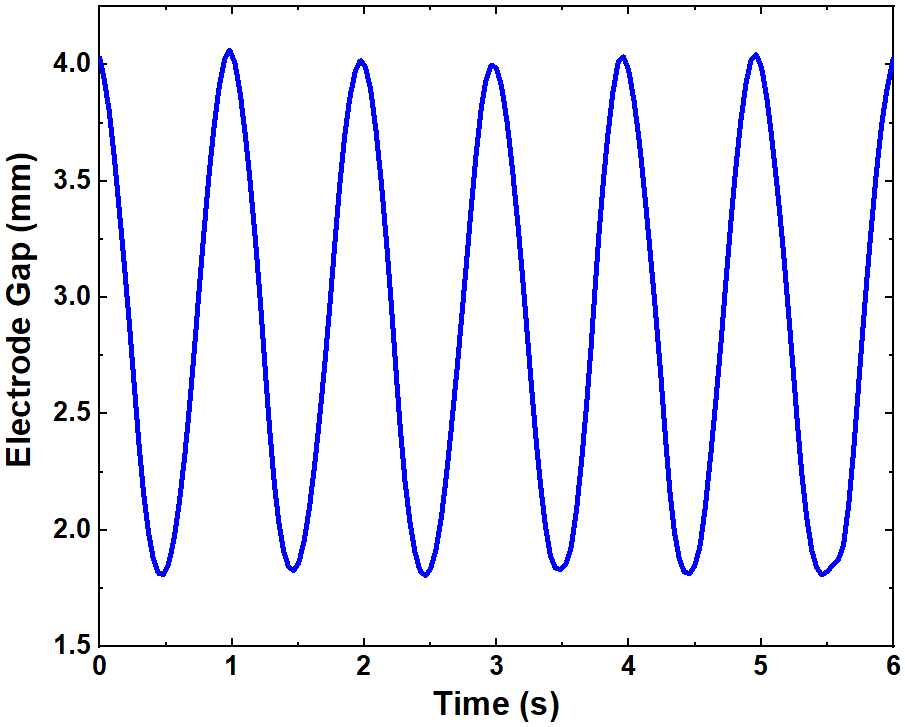


**Figure S6.** A representative plot of electrode position over time extracted from slow-motion video. Image data was processed using “ImageJ” software.
